# Residual ST-segment elevation to predict long-term clinical and CMR-derived outcomes in STEMI

**DOI:** 10.1038/s41598-022-26082-5

**Published:** 2022-12-17

**Authors:** Héctor Merenciano-González, Víctor Marcos-Garcés, Jose Gavara, Ana Pedro-Tudela, Maria P. Lopez-Lereu, Jose V. Monmeneu, Nerea Perez, Cesar Rios-Navarro, Elena de Dios, Ana Gabaldón-Pérez, Cristina Albiach, Paolo Racugno, Clara Bonanad, Joaquim Canoves, Francisco J. Chorro, Vicente Bodi

**Affiliations:** 1 Department of Cardiology, Hospital Clínico Universitario de Valencia, Valencia, Spain; 2 Health Research Institute - Instituto de Investigación Sanitaria del Hospital Clínico Universitario de Valencia (INCLIVA), Valencia, Spain; 3 Center for Biomaterials and Tissue Engineering, Universitat Politècnica de València, Valencia, Spain; 4 Department of Medicine, Faculty of Medicine and Odontology, University of Valencia, Valencia, Spain; 5 Cardiovascular Magnetic Resonance Unit, ASCIRES Biomedical Group, Valencia, Spain; 6 Centro de Investigación Biomédica en Red de Enfermedades Cardiovasculares (CIBER-CV), Madrid, Spain

**Keywords:** Cardiology, Prognostic markers, Risk factors

## Abstract

Residual ST-segment elevation after ST-segment elevation myocardial infarction (STEMI) has traditionally been considered a predictor of left ventricular (LV) dysfunction and ventricular aneurism. However, the implications in terms of long-term prognosis and cardiac magnetic resonance (CMR)-derived structural consequences are unclear. A total of 488 reperfused STEMI patients were prospectively included. The number of Q wave leads with residual ST-segment elevation > 1 mm (Q-STE) at pre-discharge ECG was assessed. LV ejection fraction (LVEF, %) and infarct size (IS, % of LV mass) were quantified in 319 patients at 6-month CMR. Major adverse cardiac events (MACE) were defined as all-cause death and/or re-admission for acute heart failure (HF), whichever occurred first. During a mean follow-up of 6.1 years, 92 MACE (18.9%), 39 deaths and 53 HF were recorded. After adjustment for baseline characteristics, Q-STE (per lead with > 1 mm) was independently associated with a higher risk of long-term MACE (HR 1.24 [1.07–1.44] per lead, *p* = 0.004), reduced (< 40%) LVEF (HR 1.36 [1.02–1.82] per lead, *p* = 0.04) and large (> 30% of LV mass) IS (HR 1.43 [1.11–1.85] per lead, *p* = 0.006) at 6-month CMR. Patients with Q-STE ≥ 2 leads (n = 172, 35.2%) displayed lower MACE-free survival, more depressed LVEF, and larger IS at 6-month CMR (*p* < 0.001 for all comparisons). Residual ST-segment elevation after STEMI represents a universally available tool that predicts worse long-term clinical and CMR-derived structural outcomes.

## Introduction

Despite the spectacular improvement in prognosis of patients with ST-segment elevation myocardial infarction (STEMI) during recent decades^1^, the short- and long-term risk of adverse outcome after the acute event is considerable^2^ and early risk stratification is recommended^3,4^. Systematic echocardiography performed before discharge should be recommended given the valuable prognostic information yielded by left ventricular (LV) ejection fraction (LVEF)^5,6^.

Electrocardiogram (ECG) is the paradigmatic non-invasive, inexpensive and universally available tool for risk stratification after STEMI. ST-segment resolution is a well-established predictor of coronary reperfusion and prognosis^7,8^. Residual ST-segment elevation after STEMI has conventionally been interpreted as predicting LV dysfunction and aneurysm^9,10^. More recently it has also been associated with more severe structural consequences in cardiac magnetic resonance (CMR) performed soon after STEMI^11,12^. Presence of Q waves on the presenting ECG or its development after STEMI have also been associated with worse clinical outcomes^13–15^ and more adverse early CMR-derived structural parameters^16,17^.

However, the contribution of pre-discharge ECG to predict long-term adverse events and the structural consequences in CMR performed late after STEMI is unclear. The aim of this study was to evaluate the relationship of combined Q wave and residual ST-segment elevation (Q-STE) with long-term cardiovascular events and CMR-derived LVEF and infarct size (IS).

## Methods

From 2012 to 2017, discharged STEMI patients treated with percutaneous coronary intervention were referred to a specific STEMI outpatient clinic, managed following current recommendations^3^ and scheduled to undergo CMR 6 months after infarction. Out of 488 prospectively recruited patients, 6-month CMR was performed in 319. The exclusion criteria were death (n = 9) or severe clinical instability (n = 7) before 6-month CMR. Unavailable or incomplete CMR studies (n = 49) or any contraindications to CMR (n = 17) were also criteria for exclusion. Other reasons for non-performance of 6-month CMR were medical decision (n = 58) or patient rejection (n = 29). The final study group therefore comprised 488 patients for evaluating the relationship between residual ST-segment elevation and long-term cardiovascular events, and 319 to evaluate the association of residual ST-segment elevation with LVEF and IS at 6-month CMR. The patient flowchart can be consulted in Supplementary Fig. S1.

This study is part of an ongoing STEMI registry of which several analyses have been previously reported^6,10,11,18,19^. The study protocol conforms to the principles of the Declaration of Helsinki and was approved by the local Human Research Ethics Committee. Patient informed consent was obtained.

Patient characteristics including Killip class at admission, peak creatine kinase MB mass, Thrombolysis in Myocardial Infarction (TIMI) flow grade in the culprit artery (before and after reperfusion) and the Global Registry of Acute Coronary Events (GRACE) and TIMI scores were recorded.

### ECG analysis

Standard 12-lead electrocardiogram (ECG) was recorded in all patients at admission, at 90 min, 6, 24, 48, and 96 h after primary percutaneous coronary intervention (pPCI), and at pre-discharge. We used a standard calibration of the ECG (10 mm/mV amplification and paper speed of 25 mm/s). ECG data was retrospectively evaluated by an independent observer blinded to clinical and angiographic data.

ST-segment elevation (STE) was measured manually in every lead 20 ms after the J-point, in accordance with previous studies^11,12^. Maximum and minimum sum of ST-segment elevation (sum-STE) before and after pPCI and at any time were quantified in mm and calculated in V1 to V6, I and aVL leads for anterior infarction and II, III, aVF, V5 and V6 for non-anterior infarction. ST-segment resolution (STR) was defined as the percentage reduction in the sum-STE from baseline (ECG on admission) to 90 min after pPCI. Finally, the number of leads with Q wave and residual STE > 1 mm (Q-STE) at pre-discharge ECG were registered.

In the present study, we focused our analyses on the impact of residual ST-segment elevation (as derived from the number of leads with Q-STE at pre-discharge ECG). The rationale underpinning this assessment was as follows: (1) Residual ST-segment elevation in Q wave leads has been previously used and validated by our group as a marker of short-term prognostic and structural outcomes after STEMI^10,11^. (2) In the hierarchical multivariable model, Q-STE emerged as the ECG index most potently associated with the pre-defined clinical and CMR-derived endpoints.

Q-STE ≥ 2 leads was used for dichotomic analyses. This approach uses the best Youden index-derived cut-off point applied to the receiver operating characteristic analysis of Q-STE to predict clinical and structural endpoints. This same definition has been previously used by our group to predict the short-term consequences of STEMI^10,11^.

### Echocardiography

All patients were studied with pre-discharge (5 ± 2 days post-STEMI) echocardiography, carried out by local cardiologists who quantified parameters and prospectively included the data in the database. LVEF (%), LV end-diastolic volume (mL) and LV end-systolic volume (mL) were assessed using the biplane method of disks (modified Simpson’s rule). Tricuspid annular plane systolic excursion (mm), as a proxy of right ventricle function, was measured in the apical 4-chamber view by means of M-mode. A wave velocity (m/s), E wave velocity (m/s), and left atrium diameter (mm) were also recorded.

### Cardiac magnetic resonance imaging

Six-month CMR (197 [181–232] days) was performed in a subgroup of 319 patients, following the exclusion criteria depicted in Supplementary Fig. S1.

Patients were examined with a 1.5 T system (Sonata Magnetom, Siemens, Erlangen, Germany) according to a previously described standard protocol^6,18^. Images were acquired by phased-array body surface coil during breath-holds and were ECG triggered. Studies were interpreted by local cardiologists specialized in CMR imaging with > 15 years of experience.

Cine images were acquired in two-, three-, and four-chamber views, and in short-axis views using a steady-state free precession sequence (repetition time/echo time: 2.8/1.2 ms; flip angle: 58 degrees; matrix: 256 × 300; field of view: 320 × 270 mm; slice thickness: 7 mm).

LV end-diastolic volume index (LVEDVI, mL/m2), end-systolic volume index (LVESVI, mL/m2) and LVEF (%) were quantified by manual planimetry of endocardial and epicardial borders in short-axis view cine images.

Late gadolinium enhancement imaging was performed 10 min after administering gadolinium-based contrast in the same locations as in the cine images using a segmented inversion recovery steady-state free precession sequence (repetition time/echo time: 750/1.26 ms; flip angle: 45 degrees; matrix: 256 × 184; field of view: 340 × 235 mm; slice thickness: 7 mm). Inversion time was adjusted to nullify normal myocardium. IS was measured by manual planimetry and defined as percentage of LV mass showing late gadolinium enhancement.

### Clinical endpoint

The primary endpoint of the study was MACE (major adverse cardiovascular events), defined as all-cause mortality or re-admission for acute heart failure, whichever occurred first. Current criteria for acute heart failure were used^20^. Clinical cardiologists prospectively adjudicated events by periodic electronic regional health system registry review.

### Structural endpoints

We measured two adverse structural endpoints at 6-month CMR: LVEF < 40% was used to define patients with reduced LV systolic function at chronic phase, while IS > 30% of LV mass was used to select patients with large myocardial infarction. The deleterious prognostic effects of these indexes and the respective cut-off points applied have already been validated by our study group in the same patient series^6,18,21^.

### Statistical analysis

The one-sample Kolmogorov–Smirnov test was used to test normal data distribution. For continuous parametric variables, data are expressed as mean ± standard deviation and analysed by Student’s t test. Continuous non-parametric variables are shown as median plus interquartile range and compared with Mann–Whitney U test. Qualitative variables are presented as percentages and compared by chi-square test or Fisher’s exact test.

Univariate analyses were performed to identify baseline, echocardiographic and ECG variables associated with clinical (MACE) and structural (LVEF < 40% and IS > 30% at 6-month CMR) endpoints.

The association of baseline, echocardiographic and ECG variables before discharge with time to first MACE was assessed by means of multivariable Cox proportional hazard regression models. Results are presented as hazard ratio (HR) and 95% confidence interval (CI). Variables with *p* value < 0.1 in univariate analysis were included as cofactors in multivariate analysis. Hierarchical models were used to avoid overfitting of variables. Model 1 included only baseline characteristics. In Model 2, variables of Model 1 plus echocardiographic indices were used. In Model 3 (final model), variables of Model 2 plus ECG indices were tested.

A multivariate binary logistic regression model using the same methodology was performed to assess the association of variables with 6-month CMR structural (LVEF < 40% and IS > 30% at 6-month CMR) endpoints.

The SPSS statistical package version 20.0 (SPSS Inc., Chicago, Illinois) and STATA version 9.0 (StataCorp, College Station, Texas) were used for statistical analysis. Statistical significance was set at *p* < 0.05.

### Ethics declaration

The study protocol was approved by the Drug Research Ethics Committee of the Hospital Clínico Universitario of Valencia. Patient informed consent was obtained.

## Results

### Characteristics of the cohort

We included 488 patients in our registry. Baseline characteristics are displayed in Table 1. Mean age was 59 ± 12 years, 80.3% were male, and smoking was the most prevalent cardiovascular risk factor. Multivessel disease was present in 25.4%, the most frequent infarct localization was anterior (50.4%) and final TIMI 3 flow grade after reperfusion was restored in 91.2% of patients. Median time to revascularization was 190 min.

**Table 1:** Baseline characteristics of the cohort.

	All patients (n = 488)	MACE		*p*
		Yes (n = 92)	No (n = 396)	
Age (years)	58.85 ± 12.43	65.63 ± 12.58	57.28 ± 11.86	< 0.001
Male sex	392 (80.3)	68 (73.9)	324 (81.8)	0.1
Smoker	279 (57.2)	45 (48.9)	234 (59.1)	0.08
Hypertension	229 (46.9)	56 (60.9)	173 (43.7)	0.003
Hypercholesterolemia	212 (43.4)	43 (46.7)	169 (42.3)	0.479
Diabetes mellitus	96 (19.7)	22 (23.9)	74 (18.7)	0.25
Previous CAD	37 (7.5)	13 (14.1)	24 (6)	0.01
**Killip class**				
I	419 (85.8)	67 (72.8)	352 (88.9)	< 0.001
≥ II	69 (14.1)	25 (27.2)	44 (11.1)	
GRACE risk score	141.1 ± 28.44	161.32 ± 32.38	136.4 ± 25.26	< 0.001
Heart rate (bpm)	79.52 ± 20.35	84.78 ± 22.71	78.3 ± 19.59	0.006
Systolic pressure (mmHg)	130.42 ± 29.66	128.45 ± 38.73	130.88 ± 29.18	0.479
**Localization**				
Anterior	246 (50.4)	60 (65.2)	186 (47)	0.002
Inferior	190 (38.9)	29 (31.5)	161 (40.6)	
Lateral	52 (10.7)	3 (3.3)	49 (12.4)	
Multivessel disease	124 (25.4)	34 (36.9)	90 (22.7)	0.005
Time to revascularization (min)	190 [130–300]	220 [150–402]	180 [120–300]	0.01
**TIMI flow grade before pPCI**				
0	231 (47.3)	45 (48.9)	186 (47)	0.71
1	33 (6.8)	8 (8.7)	25 (6.3)	
2	55 (11.3)	8 (8.7)	47 (11.9)	
3	169 (34.6)	31 (33.7)	138 (34.8)	
**TIMI flow grade after pPCI**				
0	9 (1.8)	4 (4.3)	5 (1.3)	0.012
1	3 (0.6)	1 (1.1)	2 (0.5)	
2	31 (6.4)	11 (12)	20 (5)	
3	445 (91.2)	76 (82.6)	370 (93.4)	
**Medical treatment at discharge**				
Dual antiplatelet therapy	456 (93.4)	85 (92.4)	371 (93.7)	0.64
Oral anticoagulation	67 (13.7)	15 (16.3)	52 (13.1)	0.41
Beta blockers	359 (73.6)	59 (64.1)	300 (75.8)	0.026
Angiotensin-converting-enzyme inhibitors	262 (53.7)	44 (47.8)	218 (55.1)	0.25
Angiotensin receptor blockers	114 (23.4)	23 (25)	91 (23)	0.68
Mineralocorticoid receptor antagonist	55 (11.3)	24 (26.1)	31 (7.8)	< 0.001
Statins	429 (87.9)	78 (84.8)	351 (88.6)	0.29
Diuretics	63 (12.9)	30 (32.6)	33 (8.3)	< 0.001

Categorical variables are presented as a number (percentage). Continuous parametric variables are presented as mean ± standard deviation. Continuous non-parametric variables are presented as median [interquartile range].

*bpm* Beats per minute, *CAD* Coronary artery disease, *GRACE* Global Registry of Acute Coronary Events, *MACE* Major adverse cardiovascular events, *pPCI* Primary percutaneous coronary intervention, *TIMI* Thrombolysis in myocardial infarction.

Pre-discharge echocardiographic and ECG variables are shown in Table 2. Mean LVEF was 54.9 ± 10.9%. Q-STE ≥ 2 leads was detected in 172 patients (35.2%).

**Table 2:** Echocardiographic and ECG variables before discharge.

	All patients (n = 488)	MACE		*p*
		Yes (n = 92)	No (n = 396)	
**Echocardiographic variables**				
LVEF (%)	54.93 ± 10.92	48.08 ± 13.29	56.61 ± 9.55	< 0.001
LV end-diastolic volume (mL)	108.13 ± 36.1	119.5 ± 44.32	104.84 ± 32.89	0.11
LV end-systolic volume (mL)	53.45 ± 23.5	68.86 ± 29.06	49 ± 19.66	0.002
TAPSE (mm)	20 [18–23]	19 [18–22.5]	20 [19–24]	0.86
E wave velocity (m/s)	0.72 ± 0.21	0.8 ± 0.33	0.7 ± 0.17	0.11
A wave velocity (m/s)	0.74 ± 0.19	0.79 ± 0.21	0.73 ± 0.19	0.13
Left atrium diameter (mm)	35 [32–39]	38 [33–42]	35 [31–38]	0.008
**ECG variables**				
Maximum sum-STE (mm)	11.28 ± 8.17	13.14 ± 9.19	10.85 ± 7.87	0.016
Minimum sum-STE (mm)	2.6 ± 3.01	3.11 ± 3.05	2.48 ± 2.99	0.067
Sum-STE before pPCI (mm)	11.72 ± 8.66	12.75 ± 9.25	11.5 ± 8.53	0.379
Sum-STE after pPCI (mm)	4.96 ± 4.64	5.51 ± 3.74	4.86 ± 4.83	0.469
ST resolution (%)	74.76 ± 26.73	71.67 ± 26.15	75.48 ± 26.84	0.22
Q wave (n of leads)	3 [2, 3]	3 [2–4]	3 [1–3]	< 0.001
Q-STE (n of leads)	1 [0–2]	1 [0–3]	1 [0–2]	0.003
**Q-STE**				
0–1 leads	316 (64.8)	47 (51)	269 (67.9)	0.002
≥ 2 leads	172 (35.2)	45 (49)	127 (32.1)	

Categorical variables are presented as a number (percentage). Continuous parametric variables are presented as mean ± standard deviation. Continuous non-parametric variables are presented as median [interquartile range].

*LV* Left ventricular, *LVEF* Left ventricular ejection fraction, *MACE* Major adverse cardiovascular events, *pPCI* Primary percutaneous coronary intervention, *Q-STE* Q wave and residual STE > 1 mm, *Sum-STE* Sum of ST-segment elevation, *TAPSE* Tricuspid annular plane systolic excursion.

E and A wave velocities were not considered for analyses in patients with atrial fibrillation at the time of echocardiography.

### Association of ECG variables with MACE

During a mean 6.1-year follow-up, we registered 92 (18.9%) cases of MACE (39 all-cause deaths and 53 re-admissions for acute heart failure). In hierarchical multivariable analysis, the number of leads with Q-STE > 1 mm at pre-discharge ECG was the most potent ECG predictor of MACE (HR 1.24 [1.07–1.44] per lead, *p* = 0.004). Hypertension, previous CAD, GRACE, and echocardiographic LVEF were also independently associated with MACE (Table 3). After adjustment, other ECG variables such as maximum and minimum sum-STE were not independently associated with MACE in the multivariable model.

**Table 3:** Predictors of MACE on multivariable analysis.

Variable	Model 1: Baseline characteristics		Model 2: Baseline characteristics + echocardiography		Model 3: Baseline characteristics + echocardiography + ECG	
	Hazard Ratio [95% CI]	*p*	Hazard ratio [95% CI]	*p*	Hazard ratio [95% CI]	*p*
**Baseline characteristics**						
Age (years)	1.02 [0.99–1.05]	0.19	–	–	–	–
Smoker	1.49 [0.87–2.57]	0.15	–	–	–	–
Hypertension	1.63 [1.06–2.51]	0.03	2 [1.19–3.38]	0.01	2 [1.2–3.34]	0.008
Previous CAD	3.04 [1.61–5.74]	0.001	2.7 [1.34–5.45]	0.005	1.99 [1.05–3.78]	0.04
Killip class ≥ II	0.89 [0.46–1.75]	0.74	–	–	–	–
GRACE risk score	1.02 [1.02–1.03]	< 0.001	1.02 [1.01–1.03]	< 0.001	1.02 [1.01–1.03]	< 0.001
Heart rate (bpm)	1.01 [1–1.02]	0.02	1.01 [1–1.03]	0.04	1.01 [0.99–1.02]	0.11
Inferior infarction (vs. anterior infarction)	0.83 [0.52–1.34]	0.44	–	–		
Lateral infarction (vs. anterior infarction)	0.25 [0.08–0.83]	0.02	0.4 [0.2–1.05]	0.07	–	–
Multivessel disease	1.25 [0.76–2.08]	0.38	–	–	–	–
Time to revascularization (min)	1 [0.99–1]	0.13	–	–	–	–
TIMI flow grade after pPCI < 3	1.37 [0.69–2.73]	0.37	–	–	–	–
Beta blockers	0.89 [0.55–1.43]	0.63	–	–	–	–
Mineralocorticoid receptor antagonist	2.61 [1.56–4.34]	< 0.001	1.95 [1.05–3.6]	0.03	1.64 [0.9–2.99]	0.1
Diuretics	2.47 [1.48–4.13]	0.001	1.43 [0.79–2.59]	0.24	–	–
**Echocardiographic variables**						
LVEF	–	–	0.97 [0.95–0.99]	0.006	0.96 [0.94–0.98]	< 0.001
LV end-systolic volume (mL)	–	–	–*	–*	–*	–*
Left atrium diameter (mm)	–	–	1 [0.98–1.02]	0.95	–	–
**ECG variables**						
Maximum sum-STE (mm)	–	–	–	–	1.02 [0.99–1.05]	0.25
Minimum sum-STE (mm)	–	–	–	–	0.94 [0.86–1.03]	0.2
Q wave (n of leads)	–	–	–	–	1.15 [0.97–1.35]	0.11
Q-STE (n of leads)	–	–	–	–	1.24 [1.07–1.44]	0.004

*bpm* Beats per minute, *CAD* Coronary artery disease, *CI* Confidence interval, *GRACE* Global Registry of Acute Coronary Events, *LV* Left ventricular, *LVEF* Left ventricular ejection fraction, *MACE* Major adverse cardiovascular events, *Q-STE* Q wave and residual STE > 1 mm, *Sum-STE* Sum of ST-segment elevation, *TIMI* Thrombolysis in myocardial infarction.

“Model 1: Baseline characteristics” refers to the 14 baseline variables showing an association (*p* value < 0.1 in Table 1) with occurrence of MACE.

“Model 2: Baseline characteristics + echocardiography” includes variables of Model 1 independently related to MACE plus echocardiographic indices showing an association with MACE (*p* value < 0.1 in Table 2). *LV end-systolic volume (mL) was removed from multivariable analysis due to excessive collinearity (variance inflation factor > 5 and tolerance statistic < 0.2) with LVEF.

“Model 3: Baseline characteristics + echocardiography + ECG” includes variables of Model 2 independently related to occurrence of MACE plus ECG indices showing an association with MACE (*p* value < 0.1 in Table 2).

**Figure 1:**
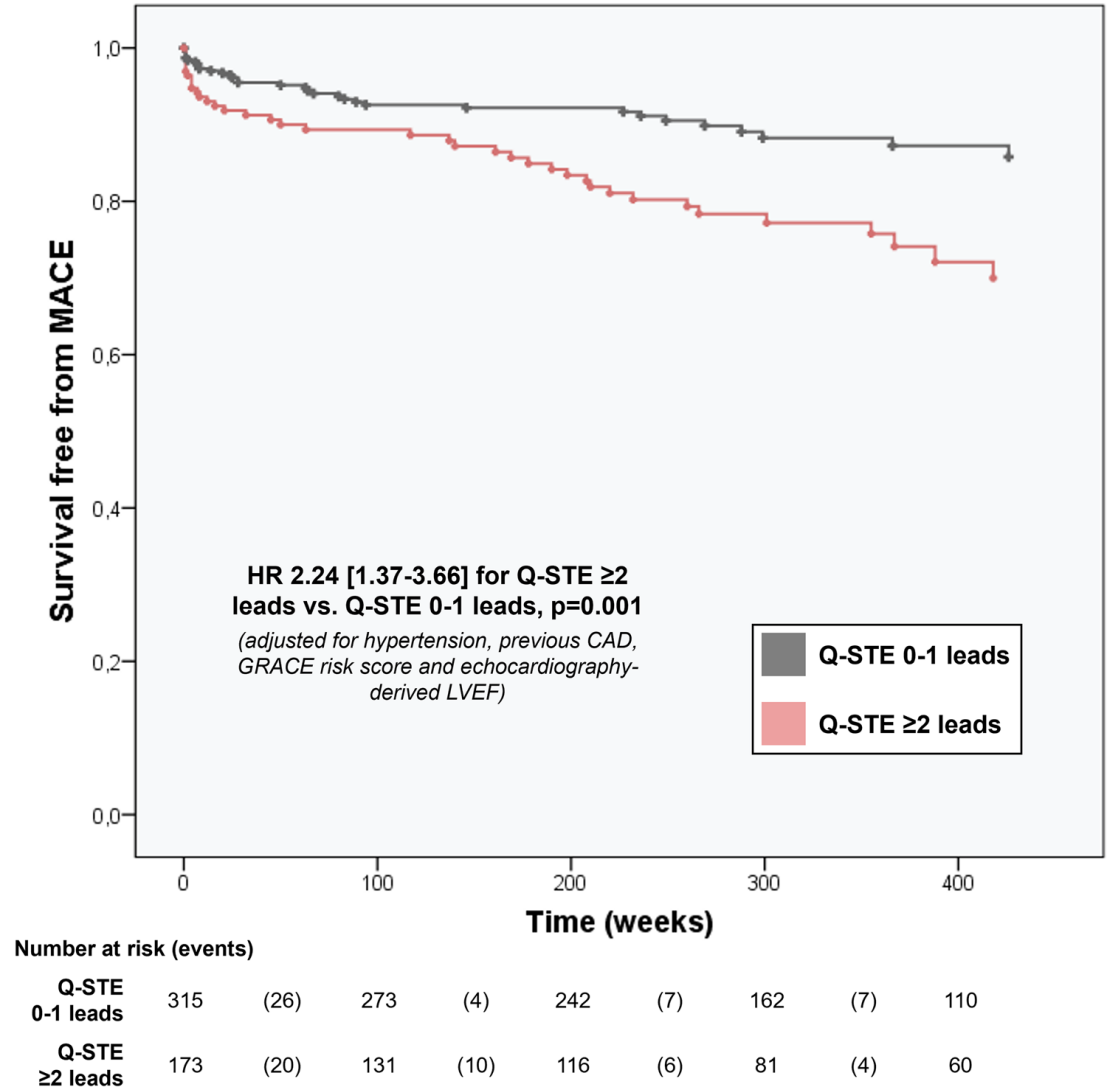
MACE-free survival by Q-STE category. Survival curves adjusted for predictors of MACE other than Q-STE (hypertension, previous CAD, GRACE risk score and echocardiography-derived LVEF). Abbreviations: CAD = coronary artery disease. GRACE = Global Registry of Acute Coronary Events. HR = hazard ratio. LVEF = Left ventricular ejection fraction. MACE = major adverse cardiovascular events. Q-STE = Q wave and residual STE > 1 mm.

Out of 172 patients with Q-STE ≥ 2 leads, 45 (26.2%) experienced MACE during follow-up, compared to 47 out of 316 (14.9%) patients with Q-STE in 0–1 leads. The adjusted survival curves are displayed in Fig. 1.

### Association of ECG variables with 6-month CMR parameters

In hierarchical multivariable analysis, the number of leads with Q-STE > 1 mm at pre-discharge ECG was the most potent ECG predictor of reduced (< 40%) LVEF and large (> 30% of LV mass) IS. After adjustment for baseline and ECG characteristics, the number of leads with Q-STE was independently and positively associated with risk of reduced (< 40%) LVEF (HR 1.36 [1.02–1.82], *p* = 0.04) and large (> 30% of LV mass) IS (HR 1.43 [1.11–1.85], *p* = 0.006) at 6-month CMR (Supplementary Tables S1, S2 and S3). Along with Q-STE, pre-discharge echocardiography LVEF was also independently associated with reduced LVEF (HR 0.86 [0.82–0.91], *p* < 0.001) and large IS (HR 0.88 [0.84–0.92], *p* < 0.001) at 6-month CMR.

**Figure 2:**
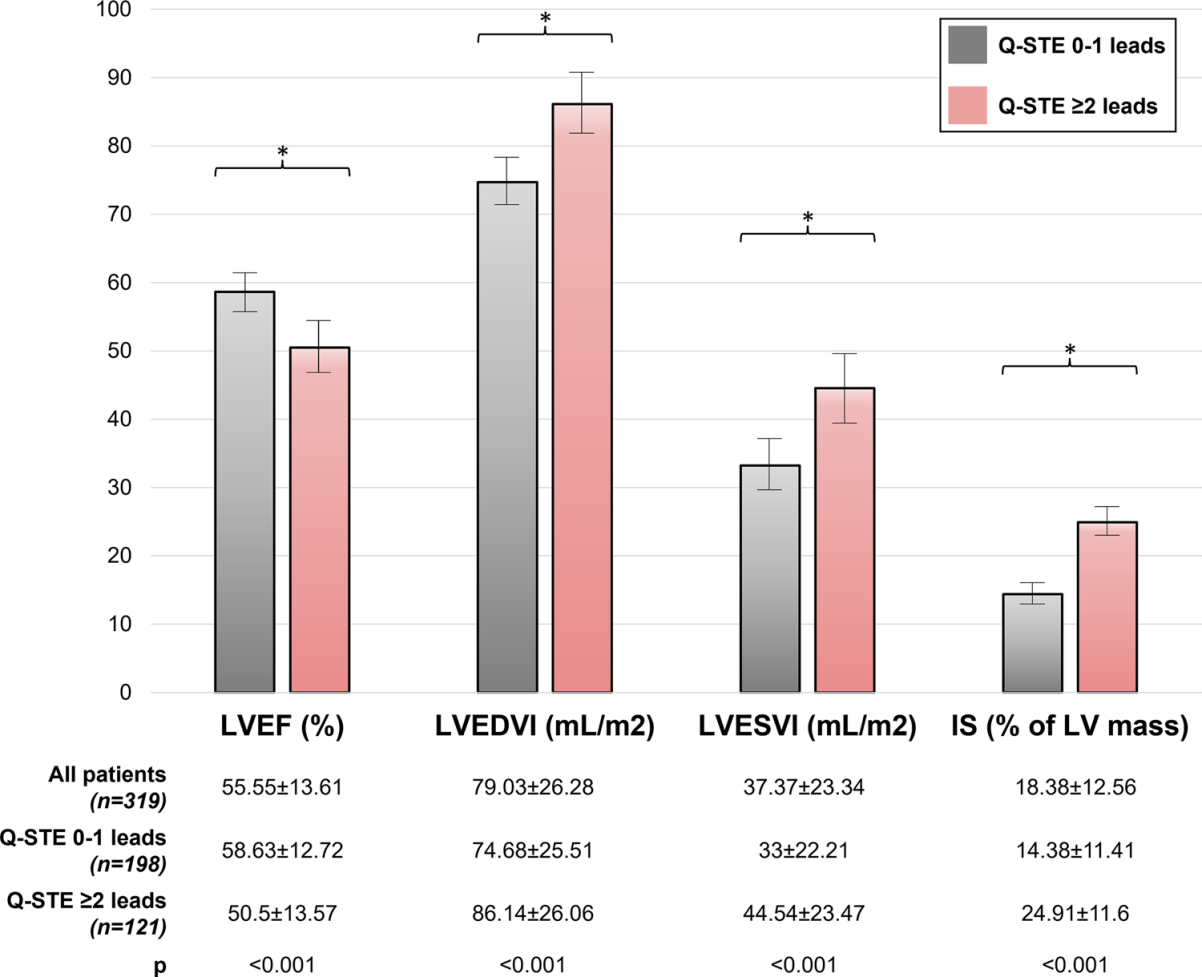
Structural changes at 6-month CMR according to Q-STE category. Variables are presented as mean ± standard deviation. * = *p* < 0.001. Abbreviations: CMR = cardiac magnetic resonance. IS = infarct size. LVEF = left ventricular ejection fraction. LVEDVI = left ventricular end-diastolic volume index. LVESVI = left ventricular end-systolic volume index. Q-STE = Q wave and residual STE > 1 mm.

**Figure 3:**
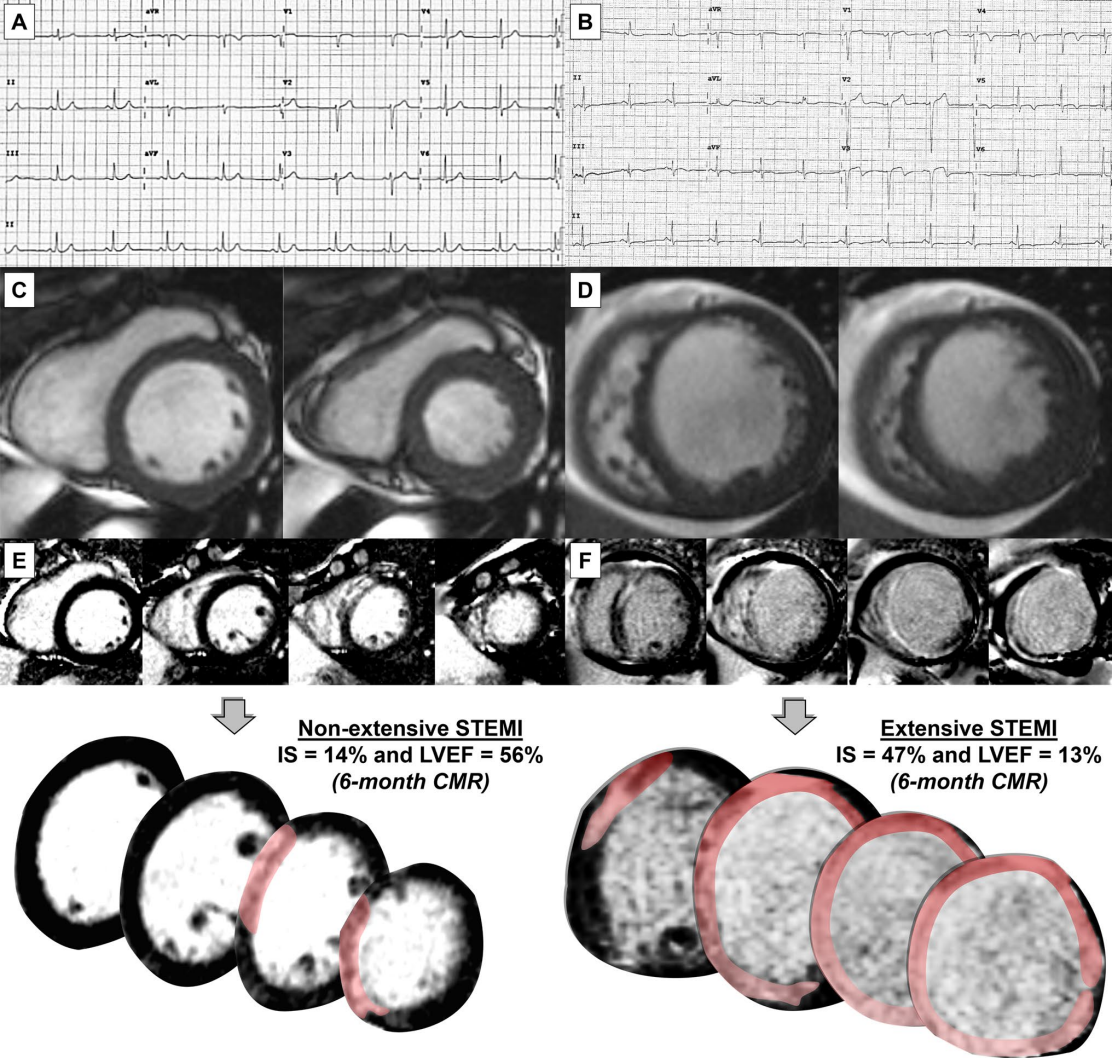
Examples of Q-STE and structural changes at 6-month CMR. Pre-discharge ECG showing no Q-STE (**A**) and Q-STE in ≥ 2 leads (**B**). Short axis CMR in diastole (left) and systole (right) indicating preserved (**C**) and reduced (< 40%, D) LVEF at 6 months. Late gadolinium enhancement imaging depicting non-extensive (**E**) and extensive (> 30% of LV mass, **F**) IS. Abbreviations: CMR = cardiac magnetic resonance. IS = infarct size. LV = left ventricular. LVEF = left ventricular ejection fraction. Q-STE = Q wave and residual STE > 1 mm.

Patients with Q-STE ≥ 2 leads displayed more reduced LVEF (50.5 ± 13.6% vs. 58.6 ± 12.7%) and more extensive IS (24.9 ± 11.6% vs. 14.4 ± 11.4%) at 6-month CMR (*p* < 0.001 for all comparisons, Figs. 2 and 3).

**Figure 4:**
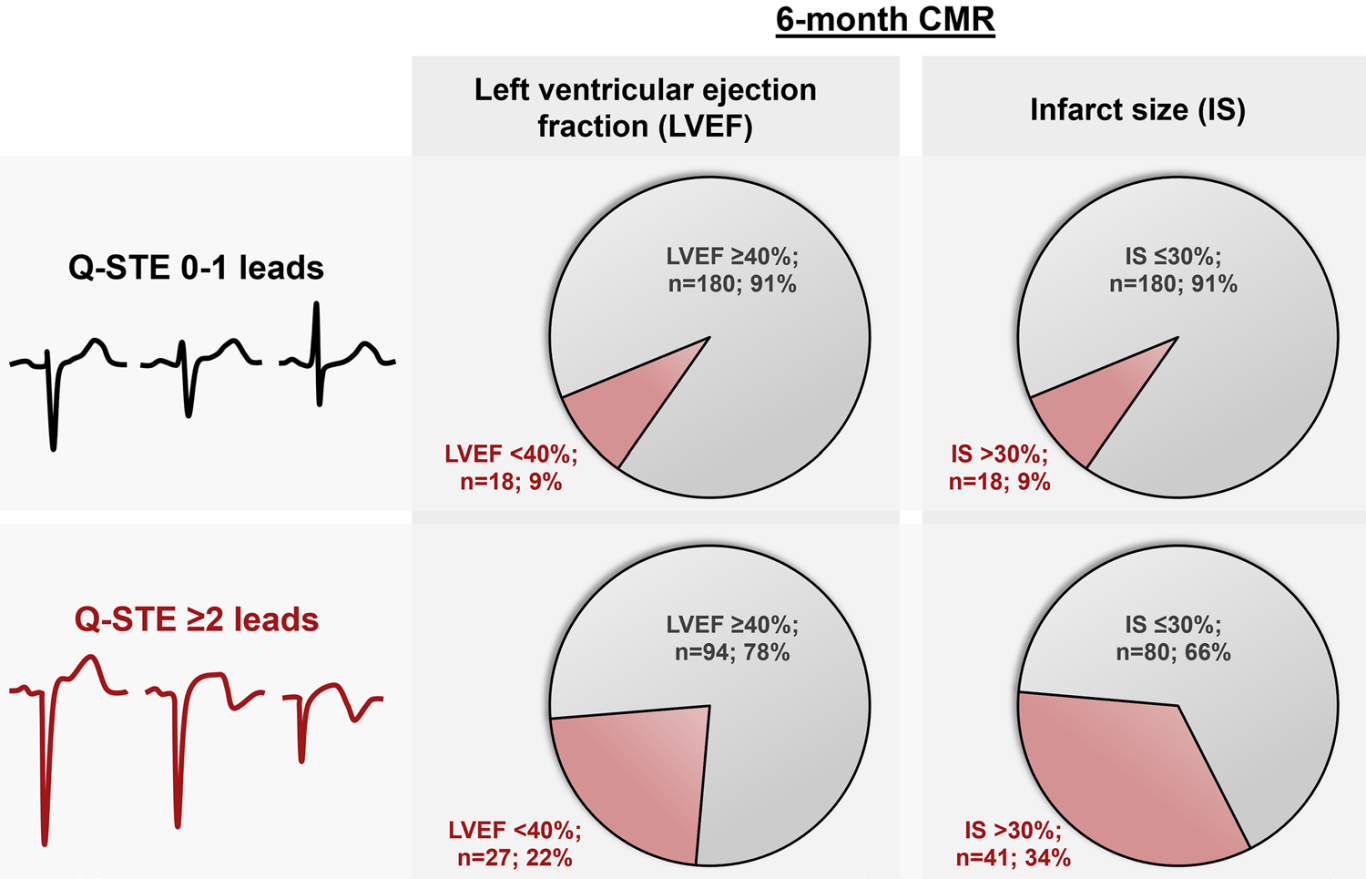
Pre-discharge Q-STE categories and structural changes at 6-month CMR. The number and percentage of patients with reduced (< 40%) LVEF and large (> 30% of LV mass) IS at 6-month CMR is shown in each Q-STE category (0–1 leads and ≥ 2 leads). Abbreviations: CMR = cardiac magnetic resonance. IS = infarct size. LV = left ventricular. LVEF = left ventricular ejection fraction. Q-STE = Q wave and residual STE > 1 mm.

Among patients with Q-STE ≥ 2 leads, 22.7% (n = 27) displayed LVEF < 40% and 33.9% (n = 41) had IS > 30% of LV mass at 6-month CMR, compared to 9.1% (n = 18) and 9.1% (n = 18) in patients with Q-STE in 0–1 leads, respectively (*p* < 0.001 for all comparisons, Fig. 4). After adjustment, patients with Q-STE ≥ 2 leads had a 3.04 [1.27–7.29]-fold and 5.83 [2.68–12.67]-fold increased probability of reduced LVEF and extensive IS at follow-up CMR compared to patients with Q-STE 0–1 leads at pre-discharge ECG (*p* = 0.01 and < 0.001, respectively).

Similar to the whole study group, in both anterior and non-anterior infarction cases, patients with Q-STE ≥ 2 leads displayed a tendency towards more altered CMR-derived structural parameters at follow-up CMR compared to those with Q-STE in 0–1 leads (Supplementary Fig. S2).

## Discussion

**Figure 5:**
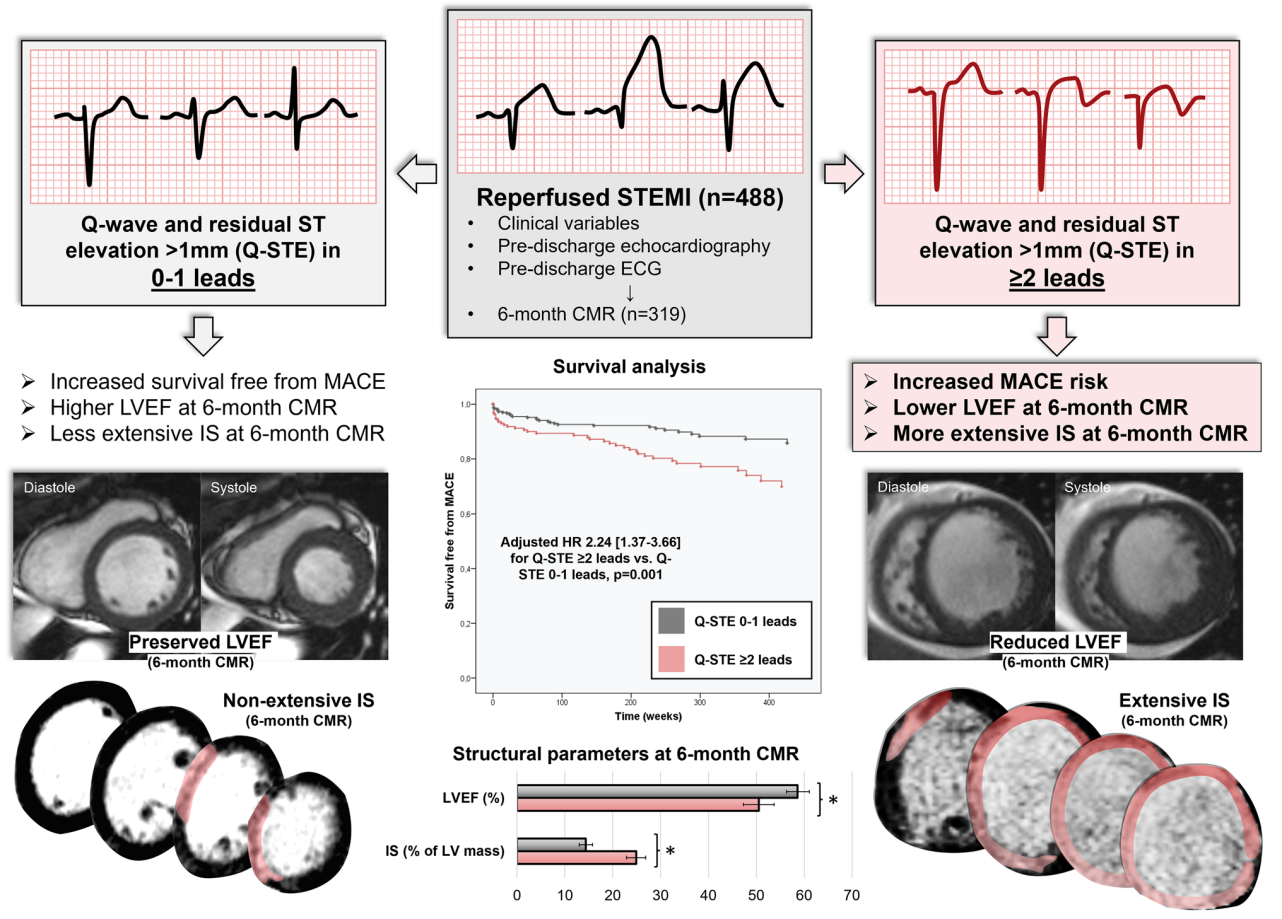
Visual summary depicting the most relevant findings of the study. Abbreviations: CMR = cardiac magnetic resonance. HR = hazard ratio. IS = infarct size. LV = left ventricular. LVEF = left ventricular ejection fraction. STEMI = ST-segment elevation myocardial infarction. Q-STE = Q wave and residual STE > 1 mm.

The main finding of the present study is that residual ST-segment elevation after reperfused STEMI contributes valuable information towards identifying a subset of patients at increased risk of long-term MACE as well as of depressed LVEF and large IS at 6-month CMR. A summary is provided in Fig. 5.

### Risk stratification after STEMI

Current management strategies in STEMI, including widespread primary percutaneous coronary intervention and optimized medical treatment, have delivered remarkable improvements in patient prognosis^1,3^. Nevertheless, the risk of MACE during the following months and years (5–20%) is non-negligible in recent registries^3^, which highlights a need for early risk stratification of STEMI patients. Several combined risk scores such as the GRACE and TIMI scores have been developed for this purpose^3^. Age is a relevant predictor of prognosis since older patients are more likely to experience all-cause death or other adverse outcomes. Nonetheless, validation of easy-to-obtain and broadly available indexes for accurate prediction of long-term clinical and structural outcomes after reperfused STEMI is still of utmost importance.

LVEF by echocardiography is the cornerstone of non-invasive imaging risk stratification after STEMI, and lower pre-discharge values have been associated with an increased long-term risk of death and re-admission for heart failure^5,22^.

CMR has become the most reliable and reproducible method for a comprehensive evaluation of the structural consequences of STEMI^23,24^, permitting highly accurate and reproducible quantification of systolic function and IS in this setting. In recent years, we and other authors have demonstrated the solid prognostic value of CMR-derived LVEF and IS^19,25,26^. Unfortunately, restrictions deriving from availability, costs and need for expertise limit the generalized use of CMR.

### Q-STE to predict long-term risk and CMR-derived IS and LVEF

Electrocardiography represents a non-invasive, lower cost, and universally attainable test that is sequentially performed in every STEMI patient. In the present study, we evaluated the long-term prognostic value of residual ST-segment elevation (as derived from the number of leads with Q-STE at pre-discharge) beyond traditional baseline and echocardiographic risk stratification. Additionally, we tested the value of this index to predict CMR-derived LVEF and IS in chronic phase after STEMI.

ST-segment resolution has been widely used as a proxy for successful coronary reperfusion^7^. Lower percentage post-reperfusion ST resolution^8,27–29^ and higher residual ST-segment elevation, either sum^30^ or residual single-lead ST-deviation^29^, have been associated with worse short-term clinical outcomes after reperfused STEMI^31^. Also, Q waves (either on the presenting ECG or developing after STEMI) have also been associated with worse clinical outcomes^13–15^ and more adverse early CMR-derived structural parameters^16,17^.

Our findings show that assessment of the number of Q wave leads with residual ST-segment elevation > 1 mm (Q-STE) at pre-discharge ECG permits immediate, robust long-term risk prediction above and beyond routine clinical and echocardiographic stratification. After adjustment, the extent of Q-STE (per number of leads) at pre-discharge appeared as an independent predictor of MACE over a 6.1-year mean follow-up. Moreover, Q-STE outperformed the rest of the collected ECG indexes for this purpose and the cut-off point proposed (Q-STE ≥ 2 leads) identified patients at high risk of MACE in a long-term perspective.

The deleterious effects of depressed LVEF and large IS are largely acknowledged, and as derived from CMR in chronic phase after STEMI, these indexes represent the cornerstone for evaluating the overall structural cardiac consequences of myocardial infarction^18,21,32–35^. Absence of ST-segment resolution has been associated with lower pre-discharge echocardiography-derived LVEF^9^. We and others have previously shown that residual ST-segment elevation associates with larger IS, more microvascular obstruction, less myocardial salvage, lower LVEF, and larger indexed LV volumes in CMR performed soon (1 week) after STEMI^10,12^ as well as more extensive IS by single-photon emission computed tomography^31^. However, the value of residual ST-segment elevation at pre-discharge ECG to predict CMR-derived LVEF and IS in chronic phase was unknown.

Our study is the first to explore in STEMI patients the association of pre-discharge ECG indexes with the long-term (6-month) CMR-derived structural consequences. Extent of Q-STE (per number of leads) was independently associated with reduced (< 40%) LVEF and large (> 30% of LV mass) IS, and the presence of Q-STE in ≥ 2 leads at pre-discharge ECG permitted good discrimination of patients at highest risk of severe structural deterioration at follow-up CMR.

### Study limitations

As a primary limitation, referral bias cannot be excluded due to the observational nature of our study and the retrospective revision of several variables. Second, this is a single-centre study and patients referred for 6-month CMR may not be entirely representative of the whole STEMI population. Prospectively designed studies could overcome these limitations. Third, several biochemical and cardiac imaging parameters that could have shown additional prognostic value were not collected. Last, changes in treatment could have influenced clinical and/or structural outcomes, requiring an in-depth analysis of the effect of therapies that is beyond the scope of the present study.

## Conclusions

Analysis of the number of Q wave leads with residual ST-segment elevation at pre-discharge ECG represents an easy-to-obtain and universally obtainable parameter which contributes rapid, valuable data on the risk of MACE, depressed LVEF and large IS at long-term follow-up. This justifies further exploration of the clinical implications of these findings, to guide more intensive follow-up, selective use of other non-invasive tests and improved therapeutic strategies.

## Supplementary Information

Supplementary Information.

## Data Availability

The datasets generated during and/or analysed during the current study are available from the corresponding author on reasonable request.
