# Supplementary material for: Residual ST-segment elevation to predict long-term clinical and CMR-derived outcomes in STEMI
Source: Sci Rep. 2022 Dec 17;12:21813. doi: 10.1038/s41598-022-26082-5 (PMC9759567; doi:10.1038/s41598-022-26082-5)

**SUPPLEMENTARY INFORMATION**

**Residual ST-segment elevation to predict long-term clinical and CMR-derived outcomes in STEMI**

Hector Merenciano-González MD^1, #^, Víctor Marcos-Garcés MD PhD^1,2, #^, Jose Gavara PhD^2,3^, Ana Pedro-Tudela^4^, Maria P. Lopez-Lereu MD PhD^5^, Jose V. Monmeneu MD PhD^5^, Nerea Perez BSc^2^, Cesar Rios-Navarro PhD^2^, Elena De Dios MSc^4,6^, Ana Gabaldón-Pérez MD^1^, Cristina Albiach MD^1^, Paolo Racugno MD^1^, Clara Bonanad MD PhD^1,2,4^, Joaquim Canoves MD PhD^1,2,4^, Francisco J. Chorro MD PhD^1,2,4,6^, Vicente Bodi MD PhD^1,2,4,6,*^

^#^These authors contributed equally.

**Author Affiliations:**

^1^Department of Cardiology, Hospital Clínico Universitario de Valencia, Valencia, Spain.

^2^Health Research Institute - INCLIVA, Valencia, Spain.

^3^Center for Biomaterials and Tissue Engineering, Universitat Politècnica de València. Valencia, Spain.

^4^Faculty of Medicine and Odontology, University of Valencia, Valencia, Spain.

^5^Cardiovascular Magnetic Resonance Unit, ASCIRES Biomedical Group, Valencia, Spain.

^6^Centro de Investigación Biomédica en Red de Enfermedades Cardiovasculares (CIBER-CV), Madrid, Spain.

**SUPPLEMENTARY TABLES**

**Supplementary Table S1. Baseline, echocardiographic and ECG characteristics according to 6-month CMR structural parameters.**

|  |  | **Reduced (<40%) LVEF on 6-month CMR** | |  | **Large (>30% of LV mass) infarct size on 6-month CMR** | |  |
| --- | --- | --- | --- | --- | --- | --- | --- |
|  | **All patients (n=319)** | **Yes (n=45)** | **No (n=274)** | **p** | **Yes (n=59)** | **No (n=260)** | **p** |
| ***Baseline characteristics*** | | | | | | | |
| **Age (years)** | 58.32±11.74 | 58.87±12.68 | 58.24±11.6 | 0.74 | 57.19±12.33 | 58.58±11.61 | 0.41 |
| **Male sex** | 261 (81.8) | 39 (86.7) | 222 (81) | 0.41 | 51 (86.4) | 210 (80.8) | 0.36 |
| **Smoker** | 193 (60.5) | 31 (68.9) | 162 (59.1) | 0.25 | 38 (64.4) | 155 (59.6) | 0.56 |
| **Hypertension** | 147 (46.1) | 21 (46.7) | 126 (46) | 1 | 28 (47.5) | 119 (45.8) | 0.89 |
| **Hypercholesterolemia** | 131 (41.1) | 24 (53.3) | 107 (39.1) | 0.08 | 27 (45.8) | 104 (40) | 0.46 |
| **Diabetes mellitus** | 56 (17.6) | 11 (24.4) | 45 (16.4) | 0.21 | 12 (20.3) | 44 (16.9) | 0.57 |
| **Previous CAD** | 14 (4.4) | 2 (4.4) | 12 (4.4) | 1 | 3 (5.1) | 11 (4.2) | 0.73 |
| **Killip class** |  |  |  |  |  |  |  |
| **I** | 276 (86.5) | 34 (75.6) | 242 (88.3) | 0.03 | 45 (76.3) | 231 (88.8) | 0.2 |
| **≥II** | 43 (13.5) | 11 (24.4) | 32 (11.7) |  | 14 (23.7) | 29 (11.2) |  |
| **GRACE risk score** | 141.04±26.54 | 152.6±29.95 | 139.14±25.5 | 0.002 | 148.22±29.04 | 139.41±25.72 | 0.02 |
| **Heart rate (bpm)** | 80.87±20.54 | 90.11±22.26 | 79.36±19.88 | 0.001 | 88.88±21.71 | 79.06±19.86 | 0.001 |
| **Systolic pressure (mmHg)** | 129.49±27.55 | 120.69±23.26 | 130.93±27.96 | 0.02 | 123.2±27.71 | 130.91±27.37 | 0.05 |
| **Localization** |  |  |  |  |  |  |  |
| **Anterior** | 174 (54.7) | 37 (82.2) | 137 (50.2) | <0.001 | 56 (94.9) | 118 (45.6) | <0.001 |
| **Inferior** | 119 (37.4) | 5 (11.1) | 114 (41.8) |  | 3 (5.1) | 116 (44.8) |  |
| **Lateral** | 25 (7.9) | 3 (6.7) | 22 (8.1) |  | 0 | 25 (9.7) |  |
| **Multivessel disease** | 76 (23.9) | 18 (40) | 58 (21.2) | 0.01 | 17 (28.8) | 59 (22.8) | 0.32 |
| **Time to revascularization (min)** | 190 [130-300] | 240 [150-450] | 185 [128-300] | 0.14 | 200 [130-399] | 195 [129-307.5] | 0.98 |
| **TIMI flow grade before pPCI** |  |  |  |  |  |  |  |
| **0** | 143 (45) | 22 (48.9) | 121 (44.3) | 0.01 | 31 (52.5) | 112 (43.2) | 0.18 |
| **1** | 21 (6.6) | 1 (2.2) | 20 (7.3) |  | 1 (1.7) | 20 (7.7) |  |
| **2** | 33 (10.4) | 10 (22.2) | 23 (8.4) |  | 8 (13.6) | 25 (9.7) |  |
| **3** | 121 (38.1) | 12 (26.7) | 109 (39.9) |  | 19 (32.2) | 102 (39.4) |  |
| **TIMI flow grade after pPCI** |  |  |  |  |  |  |  |
| **0** | 5 (1.6) | 2 (4.4) | 3 (1.1) | 0.002 | 0 | 5 (1.9) | 0.01 |
| **1** | 1 (0.3) | 0 | 1 (0.4) |  | 0 | 1 (0.4) |  |
| **2** | 20 (6.3) | 8 (17.8) | 12 (4.4) |  | 9 (15.3) | 11 (4.2) |  |
| **3** | 293 (91.8) | 35 (77.8) | 258 (94.2) |  | 50 (84.7) | 243 (93.5) |  |
| ***Medical treatment at discharge*** | | | | | | | |
| **Dual antiplatelet therapy** | 299 (93.7) | 40 (88.9) | 259 (94.5) | 0.18 | 55 (93.2) | 244 (93.8) | 0.77 |
| **Oral anticoagulation** | 35 (11) | 12 (26.7) | 23 (8.4) | 0.001 | 9 (15.3) | 26 (10) | 0.25 |
| **Beta blockers** | 229 (71.8) | 27 (60) | 202 (73.7) | 0.07 | 39 (66.1) | 190 (73.1) | 0.34 |
| **Angiotensin-converting-enzyme inhibitors** | 169 (53) | 25 (55.6) | 144 (52.6) | 0.75 | 30 (50.8) | 139 (53.5) | 0.77 |
| **Angiotensin receptor blockers** | 79 (24.8) | 13 (28.9) | 66 (24.1) | 0.46 | 18 (30.5) | 61 (23.5) | 0.32 |
| **Mineralocorticoid receptor antagonist** | 34 (10.7) | 12 (26.7) | 22 (8) | 0.001 | 15 (25.4) | 19 (7.3) | <0.001 |
| **Statins** | 276 (86.5) | 40 (88.9) | 236 (86.1) | 0.81 | 52 (88.1) | 224 (86.2) | 0.83 |
| **Diuretics** | 39 (12.2) | 14 (31.1) | 25 (9.1) | <0.001 | 13 (22) | 26 (10) | 0.02 |
| ***Echocardiographic variables*** | | | | | | | |
| **LVEF (%)** | 54.67±10.89 | 42.66±8.9 | 57.1±9.59 | <0.001 | 44.1±9.49 | 57.01±9.75 | <0.001 |
| **LV end-diastolic volume (mL)** | 109.7±34.46 | 119.71±26.51 | 107.06±36.02 | 0.22 | 117.64±30.71 | 107.6±35.35 | 0.34 |
| **LV end-systolic volume (mL)** | 54.43±21.62 | 70.79±17.54 | 50.11±20.63 | 0.001 | 66.14±19.85 | 51.34±21.17 | 0.02 |
| **TAPSE (mm)** | 20.98±3.24 | 20.71±3.09 | 21.01±3.28 | 0.82 | 19.93±2.29 | 21.12±3.34 | 0.33 |
| **E wave velocity (m/s)** | 0.69±0.16 | 0.74±0.19 | 0.69±0.15 | 0.15 | 0.75±0.14 | 0.68±0.16 | 0.14 |
| **A wave velocity (m/s)** | 0.73±0.18 | 0.67±0.22 | 0.74±0.17 | 0.14 | 0.72±0.23 | 0.74±0.17 | 0.81 |
| **Left atrium diameter (mm)** | 35 [31-38] | 36 [32-39] | 35 [31-38] | 0.29 | 37 [33-41] | 34 [31-37] | 0.14 |
| ***ECG variables*** | | | | | | | |
| **Maximum sum-STE (mm)** | 11.43±8.22 | 15.9±8.6 | 10.69±7.94 | <0.001 | 15.25±8.25 | 10.56±7.99 | <0.001 |
| **Minimum sum-STE (mm)** | 2.56±2.93 | 4.46±3.17 | 2.25±2.78 | <0.001 | 4.64±3.55 | 2.01±2.56 | <0.001 |
| **Sum-STE before pPCI (mm)** | 13.21±10.04 | 16.36±8.36 | 12.79±10.2 | 0.21 | 17.03±8.57 | 12.52±10.17 | 0.08 |
| **Sum-STE after pPCI (mm)** | 5.37±5.23 | 7.89±4.75 | 5.03±5.22 | 0.05 | 7.47±5.76 | 4.99±5.07 | 0.06 |
| **ST resolution (%)** | 75.14±26.16 | 64.13±26.75 | 76.94±25.66 | 0.002 | 65.05±22.37 | 77.42±26.45 | 0.001 |
| **Q wave (n of leads)** | 3 [2-3] | 4 [3-4] | 3 [2-3] | <0.001 | 4 [3-4] | 3 [1-3] | <0.001 |
| **Q-STE (n of leads)** | 1 [0-2] | 2 [1-3] | 0 [0-2] | 0.002 | 2 [1-3] | 0 [0-2] | <0.001 |
| **Q-STE** |  |  |  |  |  |  |  |
| **0-1 leads** | 198 (62.1) | 18 (40) | 180 (65.7) | 0.001 | 18 (30.5) | 180 (69.2) | <0.001 |
| **≥2 leads** | 121 (37.9) | 27 (60) | 94 (34.3) |  | 41 (69.5) | 80 (30.8) |  |

Categorical variables are presented as a number (percentage). Continuous parametric variables are presented as mean ± standard deviation. Continuous non-parametric variables are presented as median [interquartile range].

Abbreviations: bpm= beats per minute. CAD= coronary artery disease. GRACE= Global Registry of Acute Coronary Events. LV= Left ventricular. LVEF= Left ventricular ejection fraction. MACE= major adverse cardiovascular events. pPCI= primary percutaneous coronary intervention. Q-STE= Q wave and residual STE > 1 mm. Sum-STE= sum of ST-segment elevation. TAPSE= Tricuspid annular plane systolic excursion. TIMI= Thrombolysis in Myocardial Infarction.

In patients with atrial fibrillation at the moment of the echocardiography E and A wave velocities were not considered for analyses.

**Supplementary Table S2. Predictors of reduced (<40%) LVEF on 6-month CMR.**

|  | **Model 1: Baseline characteristics** | | **Model 2: Baseline characteristics + echocardiography** | | **Model 3: Baseline characteristics + echocardiography + ECG** | |
| --- | --- | --- | --- | --- | --- | --- |
| **Variable** | **Hazard Ratio [95% CI]** | **p** | **Hazard Ratio [95% CI]** | **p** | **Hazard Ratio [95% CI]** | **p** |
| ***Baseline characteristics*** | | | | | | |
| **Hypercholesterolemia** | 1.31 [0.63-2.73] | 0.46 | ... | ... | ... | ... |
| **Killip class ≥II** | 1.37 [0.47-4.04] | 0.57 | ... | ... | ... | ... |
| **GRACE risk score** | 1 [0.98-1.02] | 0.93 | ... | ... | ... | ... |
| **Heart rate (bpm)** | 1.02 [1-1.04] | 0.02 | 1.03 [1.01-1.05] | 0.001 | 1.03 [1-1.05] | 0.005 |
| **Systolic pressure (mmHg)** | 0.98 [0.96-0.99] | 0.001 | 0.98 [0.96-0.99] | 0.006 | 0.98 [0.96-0.99] | 0.007 |
| **Inferior infarction (vs anterior infarction)** | 0.18 [0.06-0.5] | 0.001 | 0.28 [0.07-2.1] | 0.07 | ... | ... |
| **Multivessel disease** | 3.39 [1.62-7.12] | 0.001 | 3.81 [1.57-9.26] | 0.003 | 3.78 [1.54-9.27] | 0.004 |
| **TIMI flow grade before pPCI <3** | 1.55 [0.27-8.87] | 0.62 | ... | ... | ... | ... |
| **TIMI flow grade 0 after pPCI** | 5.45 [1.86-15.98] | 0.002 | 4.22 [0.98-17.34] | 0.06 | ... | ... |
| **Oral anticoagulation** | 1.89 [0.68-5.28] | 0.22 | ... | ... | ... | ... |
| **Beta blockers** | 0.47 [0.22-1.04] | 0.06 | ... | ... | ... | ... |
| **Mineralocorticoid receptor antagonist** | 2.66 [1.13-6.25] | 0.03 | 1.61 [0.56-4.63] | 0.38 | ... | ... |
| **Diuretics** | 2.63 [1.06-6.56] | 0.04 | 2.33 [0.72-7.57] | 0.16 | ... | ... |
| ***Echocardiographic variables*** | | | | | | |
| **LVEF** | ... | ... | 0.86 [0.82-0.9] | <0.001 | 0.86 [0.82-0.91] | <0.001 |
| **LV end-systolic volume (mL)** | ... | ... | ...* | ...* | ...* | ...* |
| ***ECG variables*** | | | | | | |
| **Maximum sum-STE (mm)** | ... | ... | ... | ... | 0.99 [0.85-1.16] | 0.91 |
| **Minimum sum-STE (mm)** | ... | ... | ... | ... | 1.08 [0.72-1.62] | 0.73 |
| **Sum-STE after pPCI (mm)** | ... | ... | ... | ... | 1.04 [0.83-1.29] | 0.76 |
| **ST resolution (%)** | ... | ... | ... | ... | 1.01 [0.96-1.07] | 0.66 |
| **Q wave (n of leads)** | ... | ... | ... | ... | 1.46 [0.87-2.46] | 0.17 |
| **Q-STE (n of leads)** | ... | ... | ... | ... | 1.36 [1.02-1.82] | 0.04 |

Abbreviations: bpm= beats per minute. CI= confidence interval. CMR= cardiac magnetic resonance. GRACE= Global Registry of Acute Coronary Events. LV= Left ventricular. LVEF= Left ventricular ejection fraction. pPCI= primary percutaneous coronary intervention. Q-STE= Q wave and residual STE > 1 mm. Sum-STE= sum of ST-segment elevation. TIMI= Thrombolysis in Myocardial Infarction.

“Model 1: Baseline characteristics” refers to the 13 baseline variables showing an association (p-value<0.1 in Supplemental Table 1) with the occurrence of reduced (<40%) LVEF on 6-month CMR.

“Model 2: Baseline characteristics + echocardiography” includes variables of Model 1 plus echocardiographic indices showing an association with reduced (<40%) LVEF on 6-month CMR (p-value<0.1 in Supplemental Table 1). *LV end-systolic volume (mL) was removed from multivariable analysis due to excessive collinearity (variance inflation factor>5 and tolerance statistic<0.2) with LVEF.

“Model 3: Baseline characteristics + echocardiography + ECG” includes variables of Model 2 plus ECG indices showing an association with reduced (<40%) LVEF on 6-month CMR (p-value<0.1 in Supplemental Table 1).

**Supplementary Table S3. Predictors of large (>30% of LV mass) infarct size on 6-month CMR.**

|  | **Model 1: Baseline characteristics** | | **Model 2: Baseline characteristics + echocardiography** | | **Model 3: Baseline characteristics + echocardiography + ECG** | |
| --- | --- | --- | --- | --- | --- | --- |
| **Variable** | **Hazard Ratio [95% CI]** | **p** | **Hazard Ratio [95% CI]** | **p** | **Hazard Ratio [95% CI]** | **p** |
| ***Baseline characteristics*** | | | | | | |
| **GRACE risk score** | 1 [0.99-1.02] | 0.6 | ... | ... | ... | ... |
| **Heart rate (bpm)** | 1.02 [0.99-1.03] | 0.06 | ... | ... | ... | ... |
| **Systolic pressure (mmHg)** | 0.98 [0.97-0.99] | 0.003 | 0.98 [0.97-1] | 0.04 | 0.87 [0.93-1.01] | 0.13 |
| **Inferior infarction (vs anterior infarction)** | 0.05 [0.02-0.18] | <0.001 | 0.04 [0-0.28] | 0.002 | 0 [0-0] | 1 |
| **TIMI flow grade 0 after pPCI** | 3.66 [1.21-11.06] | 0.02 | 1.71 [0.41-7.23] | 0.47 | ... | ... |
| **Mineralocorticoid receptor antagonist** | 2.49 [1.07-5.78] | 0.04 | 1.77 [0.61-5.15] | 0.29 | ... | ... |
| **Diuretics** | 2.54 [1.22-5.32] | 0.01 | 1.59 [0.5-5.02] | 0.43 | ... | ... |
| ***Echocardiographic variables*** | | | | | | |
| **LVEF** | ... | ... | 0.9 [0.86-0.94] | <0.001 | 0.88 [0.84-0.92] | <0.001 |
| **LV end-systolic volume (mL)** | ... | ... | ...* | ...* | ...* | ...* |
| ***ECG variables*** | | | | | | |
| **Maximum sum-STE (mm)** | ... | ... | ... | ... | 1.01 [0.81-1.26] | 0.95 |
| **Minimum sum-STE (mm)** | ... | ... | ... | ... | 1.64 [0.92-2.9] | 0.09 |
| **Sum-STE before pPCI (mm)** | ... | ... | ... | ... | 0.89 [0.69-1.13] | 0.33 |
| **Sum-STE after pPCI (mm)** | ... | ... | ... | ... | 1.08 [0.82-1.41] | 0.58 |
| **ST resolution (%)** | ... | ... | ... | ... | 1.09 [0.99-1.19] | 0.09 |
| **Q wave (n of leads)** | ... | ... | ... | ... | 1.54 [0.83-2.86] | 0.17 |
| **Q-STE (n of leads)** | ... | ... | ... | ... | 1.43 [1.11-1.85] | 0.006 |

Abbreviations: bpm= beats per minute. CI= confidence interval. CMR= cardiac magnetic resonance. GRACE= Global Registry of Acute Coronary Events. LV= Left ventricular. LVEF= Left ventricular ejection fraction. pPCI= primary percutaneous coronary intervention. Sum-STE= summatory of ST-segment elevation. Q-STE= Q wave and residual STE > 1 mm. TIMI= Thrombolysis in Myocardial Infarction.

“Model 1: Baseline characteristics” refers to the 7 baseline variables showing an association (p-value<0.1 in Supplemental Table 1) with the occurrence of large (>30% of LV mass) infarct size on 6-month CMR.

“Model 2: Baseline characteristics + echocardiography” includes variables of Model 1 plus echocardiographic indices showing an association with large (>30% of LV mass) infarct size on 6-month CMR (p-value<0.1 in Supplemental Table 1). *LV end-systolic volume (mL) was removed from multivariable analysis due to excessive collinearity (variance inflation factor>5 and tolerance statistic<0.2) with LVEF.

“Model 3: Baseline characteristics + echocardiography + ECG” includes variables of Model 2 plus ECG indices showing an association with large (>30% of LV mass) infarct size on 6-month CMR (p-value<0.1 in Supplemental Table 1).

**SUPPLEMENTARY FIGURES**

**Supplementary Figure S1. Flowchart of patients included in the study.**


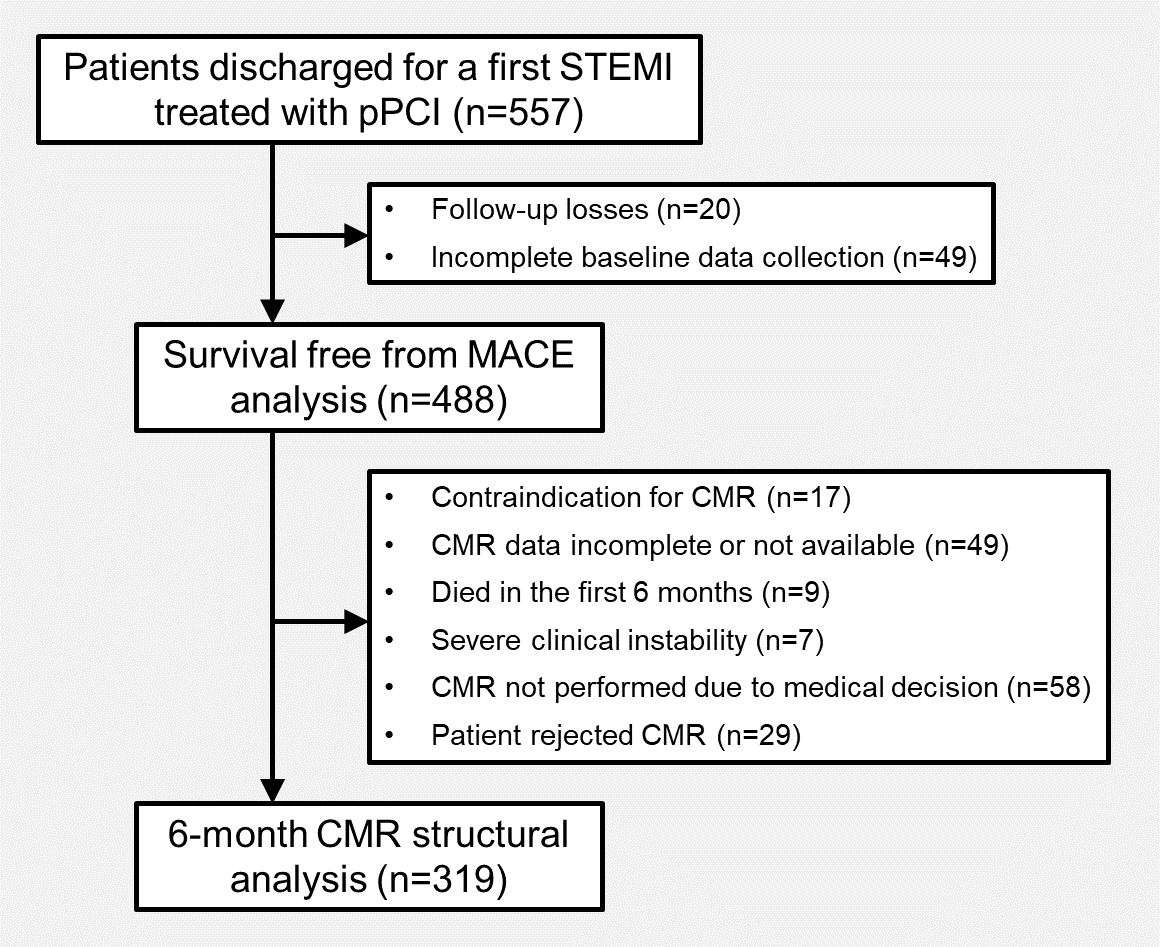


Abbreviations: CMR= cardiac magnetic resonance. MACE= major adverse cardiovascular events. pPCI= primary percutaneous coronary intervention. STEMI= ST-segment elevation myocardial infarction.

**Supplementary Figure S2. Structural changes at 6-month CMR according to Q-STE categories and infarct location (anterior vs. non-anterior).** Variables are presented as mean ± standard deviation. * = p<0.001.

Abbreviations: CMR= cardiac magnetic resonance. IS= infarct size. LV= left ventricular. LVEF= left ventricular ejection fraction. LVEDVI= left ventricular end-diastolic volume index. LVESVI= left ventricular end-systolic volume index. Q-STE= Q wave and residual STE > 1 mm.


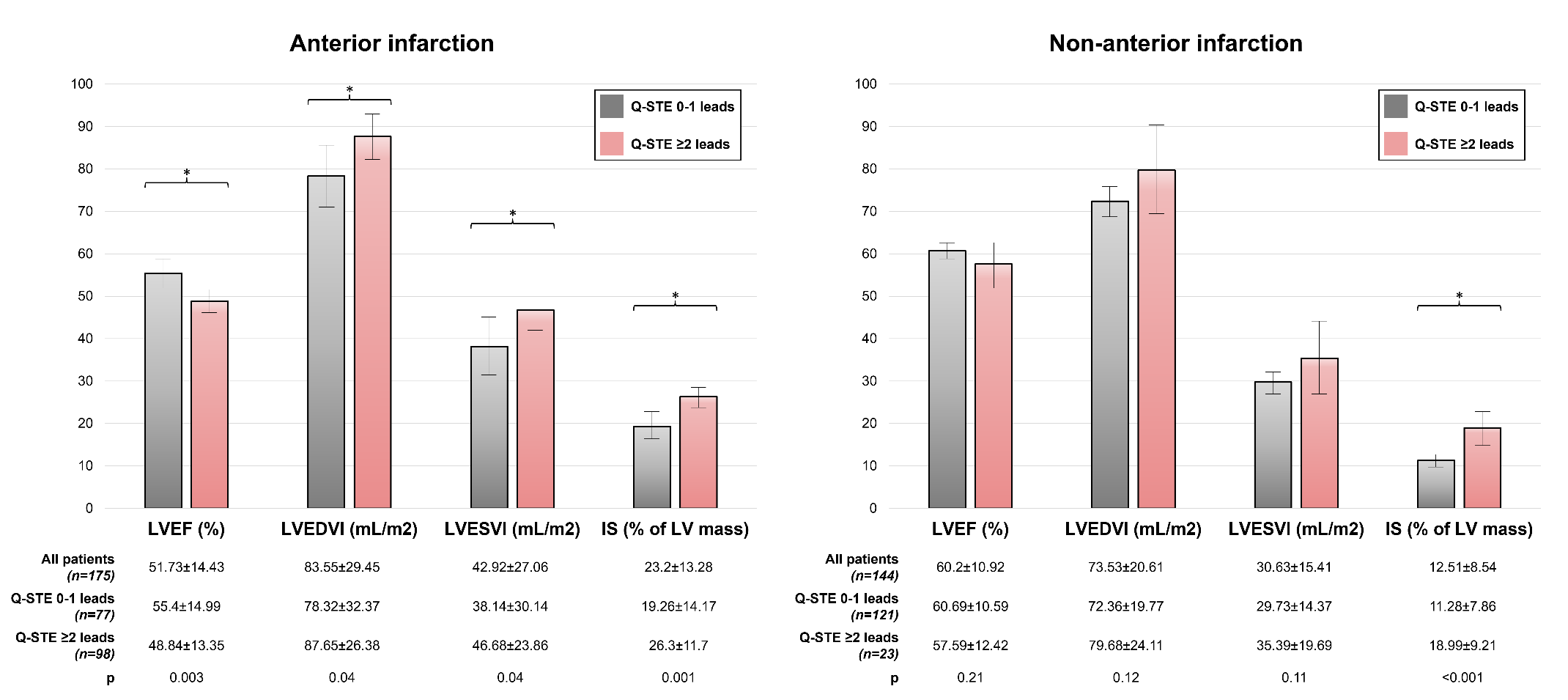

Supplement: Supplementary file 1 — Supplementary Information. [file 41598_2022_26082_MOESM1_ESM.docx]
